# The Pan-American Medical Congress

**Published:** 1893-03

**Authors:** 


### THE PAN-AMERICAN MEDICAL CONGRESS.

WE have received the "Preliminary Manifesto of the Section of Nervous and Mental Diseases" of this Congress, and are requested to state that "every effort is being made to make the meeting of the Section on Diseases of the Mind and Nervous System both scientifically profitable and socially pleasant."

A long list of officers is appended, for which we have not the space.

This Congress, notwithstanding the numerous attractions at Chicago, appears to promise excellent results.

